# Upregulated Fibulin-1 Increased Endometrial Stromal Cell Viability and Migration by Repressing EFEMP1-Dependent Ferroptosis in Endometriosis

**DOI:** 10.1155/2022/4809415

**Published:** 2022-01-28

**Authors:** Yiting Wan, Yanhua Song, Jing Chen, Jueying Kong, CanCan Gu, Jiami Huang, Ling Zuo

**Affiliations:** Department of Gynecology, Shanghai Municipal Hospital of Traditional Chinese Medicine, Shanghai University of Traditional Chinese Medicine, Shanghai 200071, China

## Abstract

Endometriosis (EMS) is a prevalent disease in women characterized by the presence of endometrial stroma and glands outside the uterus. Recent studies have showed that EMS is correlated with the resistance of endometrial stromal cells (ESCs) to ferroptosis, an iron-dependent nonapoptotic cell death. Fibulin-1 (FBLN1) is a newly identified target regulated by progesterone in the process of ESC decidualization. However, the role of FBLN1 in regulating ESC ferroptosis and EMS remains unclear. In the present study, the gene expression profiles of GSE58178, GSE23339, and GSE25628 were downloaded from the Gene Expression Omnibus (GEO) database, and the commonly differential genes were identified using Venn diagram analysis. The role of FBLN1 in ESC viability and migration was evaluated using Cell Counting Kit-8, transwell, and western blot analysis. We found that the FBLN1 expression was increased significantly in eutopic and ectopic endometrial tissues of patients with EMS compared with normal endometrium. FBLN1 overexpression in normal ESCs (NESCs) promoted cell viability and migration, whereas FBLN1 inhibition in ectopic ESCs (EESCs) decreased cell viability and migration. Furthermore, FBLN1 inhibition facilitated EESC death by triggering ferroptosis, as evidenced by increased Fe^2+^, lipid ROS, and malondialdehyde (MDA) level and decreased glutathione peroxidase 4 (GPX4) expression and glutathione (GSH) level. Mechanistically, FBLN1 interacted with EGF-containing fibulin-like extracellular matrix protein 1 (EFEMP1) and increased the protein stability of EFEMP1. More importantly, EFEMP1 silencing repressed the effect of FBLN1 on regulating EESC ferroptosis, death, and migration. Taken together, these results verify the role of the FBLN1/EFEMP1/ferroptosis pathway in the pathogenesis of EMS, and silencing of FBLN1/EFEMP1 might be an effective approach to treat EMS.

## 1. Introduction

Endometriosis (EMS) is a common gynecological disease in women characterized by the implantation of active endometrial cells outside the uterine cavity [1]. The main pathological changes of EMS are periodic bleeding of ectopic endometrium and fibrosis of surrounding tissues [2]. The incidence of EMS is as high as 35-50% in women with pain and infertility [3]. Unfortunately, EMS is underdiagnosed, with an average latency of 6.7 years from symptom onset to final diagnosis [4]. It is urgent to identify the molecular mechanism involved in the etiology of EMS to improve the diagnosis and treatment of EMS.

Ferroptosis is a newly identified mechanism of nonapoptotic cell death mediated by iron [5–8]. Ferroptosis clearly differs from other forms of cell death including apoptosis, autophagy, and necrosis. Ferroptosis is induced by an imbalance between generation and degradation of iron-dependent lipid reactive oxygen species (ROS) [9, 10]. Mounting studies have demonstrated that ferroptosis might be implicated in a variety of pathologic processes, such as neurodegenerative diseases, acute renal failure, hepatotoxicity, and cardiovascular disease [11, 12]. The role of ferroptosis in EMS has not yet been systematically described. Previous studies revealed the increased levels of iron and lipid peroxide content within low-density lipoprotein (LDL) particles in the peritoneal fluid of women with EMS compared to those without [13–15]. Iron conglomerates are typically found in endometriotic lesions in murine models of EMS and in patients with EMS [16, 17]. These data suggest that EMS is correlated with dysregulation of localized iron homeostasis that promotes lipid peroxidation and subsequent ferroptosis within the peritoneal cavity [13, 18, 19].

FBLN1 is a secretory glycoprotein that maintains the stability of the extracellular matrix (ECM) [20, 21]. FBLN1 can bind to target proteins (fibronectin, fibrinogen, angiopoietin, laminin-1, etc.) to inhibit the signal transduction, cancer cell growth, migration, and invasion [22]. Consequently, FBLN1 functions as a tumor suppressor in gastric cancer, prostate cancer, breast cancer, and ovarian cancer [23]. Besides, FBLN1 is aberrantly expressed in ovarian and breast cancer cells and has been identified as a breast cancer-restricted antigen [24]. Interestingly, FBLN1 is a newly identified target regulated by progesterone during ESC decidualization. However, the role of FBLN1 in EMS remains poorly understood.

Based on above findings, we investigated whether the expression of FBLN1 was dysregulated in ectopic endometrial tissues and whether aberrant FBLN1 was correlated with EMS. We demonstrated that FBLN1 expression was upregulated in eutopic and ectopic endometrial tissues of patients with EMS. FBLN1 silencing decreased EESC viability and migration and facilitated EESC ferroptosis by increasing the protein stability of EFEMP1.

## 2. Materials and Methods

### 2.1. Clinical Specimens

The protocol was approved by the Ethics Committee of Shanghai Municipal Hospital of Traditional Chinese Medicine, Shanghai University of Traditional Chinese Medicine (2018SHL-KYYS-19), and informed consent was obtained from all volunteers. Twelve volunteers (6 patients with ovarian EMS (1 patient in stage II, 3 patients in stage III, and 2 patients in stage IV) and 6 normal controls with fibroid or other benign gynecological disorders) were recruited to participate in the research from July 2019 to July 2020. Inclusion criteria were as follows: patients aged 22 to 45 years old and met the diagnostic criteria of EMS [25], women with regular menstrual cycles, and no hormonal medicine therapy for 3 months before surgery. Exclusion criteria were as follows: patients with primary dysmenorrheal, pelvic inflammatory disorders, reproductive system and other types of cancers, and polycystic ovary syndrome, and women with recent contraception and bacterial, fungal, or virus infection were excluded from the present study. Ectopic and paired eutopic endometrial tissues were obtained from patients with EMS at the proliferative phase of menstrual cycles. The normal endometrial tissue samples were obtained from controls undergoing surgery for fibroid or other benign gynecological disorders.

### 2.2. Primary Cell Extraction

Primary normal ESCs (NESCs) were isolated from normal endometrial tissues, and ectopic ESCs (EESCs) were obtained from ectopic endometrial tissues in patients with EMS as described previously [26]. In brief, the tissues were washed using PBS, cut into 1 mm^3^ pieces, and digested with 5% dispase enzyme and collagenase (Sigma-Aldrich, St. Louis, MO, USA) for 1 h at 37°C, continuously filtered to separate, and then treated with red blood cell lysis buffer (Sigma-Aldrich). Epithelial cells were separated by a 40 *μ*m sieve, and stromal cells were further enriched. The NESCs and EESCs were kept in DMEM contained with 10% (*v*/*v*) FBS, 1% penicillin/streptomycin, and 0.025 mg/mL of amphotericin B, in a humidified 5% CO_2_ environment at 37°C.

### 2.3. RNA Interference (RNAi) and Overexpression

FBLN1 siRNA, EFEMP1 siRNA, and control siRNA were purchased from HANBIO (Shanghai, China). The pcDNA3.1 vector was obtained from Vazyme (Nanjing, Jiangsu, China). Full-length FBLN1 was subcloned into pcDNA3.1 vector to construct recombinant plasmid pcDNA-FBLN1 to overexpress FBLN1. Before transfection, the ESCs of logarithmic growth phase were inoculated into culture dish, and the cell fusion reached 60-80% confluence on the second day. Lipofectamine™ 3000 (Invitrogen, Carlsbad, CA, USA) was applied to prepare the mixture of FBLN1-siRNA, EFEMP1 siRNA, control-siRNA, or pcDNA-FBLN1-Lipofectamine™ 3000 as the instructions. The cells were collected for 48 hours after transfection.

### 2.4. Quantitative Real-Time PCR (qPCR)

Total RNA was extracted from normal endometrial tissues and eutopic and ectopic endometrial tissues in patients with EMS with Trizol RNA extraction kit (Invitrogen). To assess the mRNA level of GPX4, total RNA was obtained from EESCs after erastin treatment and FBLN1 overexpression. The reverse transcription (RT) was carried out using SuperScript IV Reverse Transcriptase (Thermo Fisher Scientific, MA, USA). qPCR was performed on an Applied Biosystems 7300 real-time PCR system (Applied Biosystems, CA, USA) using 2x SYBR Green qPCR Master Mix (APExBIO, Houston, USA). The relative expression was calculated through the 2^-*ΔΔ*Ct^ method, and the *β*-actin gene was set as the reference gene.

### 2.5. Fluorescein Diacetate (FDA) Staining

After washing with HBSS, ESCs were incubated with 10 mL FDA solution (10 mg/mL; Sigma-Aldrich) at 37°C for 15 min. Then, the cells were observed and photographed with an inverted fluorescence microscope (Olympus Corporation, Japan). FDA-positive ratio from 10 random fields was quantified by ImageJ software.

### 2.6. Cycloheximide Chase Assay

ESCs were treated with 15 *μ*g/mL CHX for 0, 2, 4, and 8 h. Cell lysate was collected and analyzed through western blotting using antibodies against EFEMP1 and GAPDH. EFEMP1 protein band intensity at each time point was quantified and normalized to GAPDH using ImageJ software.

### 2.7. Reduced Glutathione (GSH) Assay

The glutathione assay kit (Sigma-Aldrich) was used to evaluate the relative GSH concentration in the EESC lysate as the manufacturer's protocol.

### 2.8. Iron Assay

Intracellular ferrous iron (Fe^2+^) level was detected with the iron assay kit (ab83366, Abcam) as the manufacturer's instructions.

### 2.9. Lipid Peroxidation (MDA) Assay

The concentration of malondialdehyde (MDA), one of the end products of lipid peroxidation, was detected with the lipid peroxidation assay kit (K739-100, BioVision) following the manufacturer's instructions.

### 2.10. Lipid ROS Detection

Intracellular lipid ROS levels were detected with a peroxide-sensitive fluorescent probe C11-BODIPY (Thermo Fisher Scientific) as the manufacturer's instructions. In brief, the EESCs were seeded into 6-well plates and exposed to erastin 10 *μ*M for 24 h or transfected with FBLN1 siRNA, pcDNA3.1-FBLN1, or EFEMP1 siRNA for 48 h. Then, cells were incubated with C11-BODIPY at a final concentration of 10 *μ*M in medium without FBS at 37°C for 0.5 h and washed three times using medium.

### 2.11. Immunohistochemistry (IHC)

Endometrial tissues were fixed with 10% buffered formalin for 2 days, cut into 4-6 mm slices, dehydrated by ethanol, and prepared in paraffin. Then, the slices were incubated with primary antibody against FBLN1 (1 : 200, ab211536, Abcam) overnight at 4°C. In addition, the slices were incubated with secondary antibody Goat Anti-Mouse IgG H&L (HRP) (1 : 500, ab205719, Abcam), and the DAB solution (Solarbio, Beijing, China) was applied to visually analyze the reaction products. FBLN1 IHC score was calculated as previously described [27].

### 2.12. Cell Viability

Cell viability was assessed using CCK-8 (Dojindo Laboratories, Japan) following the manufacturer's instructions. In brief, cells were seeded into 96-well plates at a density of 5 × 10^3^ cells/well to incubate for 24 h, and the cells were transfected with FBLN1 siRNA, pcDNA3.1-FBLN1, or EFEMP1 siRNA for 48 h and then treated with indicated inhibitors (ZVAD-FMK, 10 *μ*M; necrostatin-1, 10 *μ*M; ferrostatin-1, 1 *μ*M) for 24 h. After that, 10 *μ*L CCK-8 solution was added to each well and incubated for 4 h. The absorbance (450 nm) was calculated with a microplate reader (Molecular Devices, CA, USA).

### 2.13. Transwell Migration Assay

Transwell migration assay was carried out using a transwell chamber (24-well, 8 *μ*m pore size). Cells (4 × 10^4^) resuspended in 100 *μ*L of serum-free medium were seeded into the upper chamber, and the lower chamber was added with 600 *μ*L DMEM medium supplemented with 10% FBS. Transwell chamber was incubated for one day at 37°C. The nonmigrated cells were removed using cotton swabs, and the migrated cells were fixed with formaldehyde and stained with 0.1% crystal violet for 15 min.

### 2.14. Western Blot Analysis

Total protein was collected from normal endometrial tissues, eutopic and ectopic endometrial tissues with EMS, or cells with RIPA Lysis and Extraction Buffer (Thermo Scientific). Approximately 20 *μ*g of total protein was loaded on 12% sodium dodecyl sulfate-polyacrylamide gels (SDS-PAGE) and transferred to a 0.2 *μ*m PVDF membranes (Roche, Basel, Switzerland). The blots were incubated overnight at 4°C with primary antibodies against FBLN1 (1 : 1000, ab17204, Abcam), EFEMP1 (1 : 1000, ab256457, Abcam), and *β*-actin: (1 : 2000, ab8227, Abcam) and were then incubated with a goat anti-rabbit HRP-conjugated secondary antibody. The protein signals were visualized by an ECL kit (Solarbio).

### 2.15. Statistical Analyses

Data are represented as the mean ± SD from at least three independent experiments. The differences between two groups were conducted by Student's *t*-test or one-way ANOVA followed by the Scheffé test. *p* < 0.05 was considered to indicate a statistically significant difference. All statistical analyses were performed using the SPSS program (version 20.0; IBM, Somers, NY, USA).

## 3. Results

### 3.1. FBLN1 Expression Was Upregulated in Eutopic and Ectopic Endometrial Tissues with EMS

**Figure 1:**
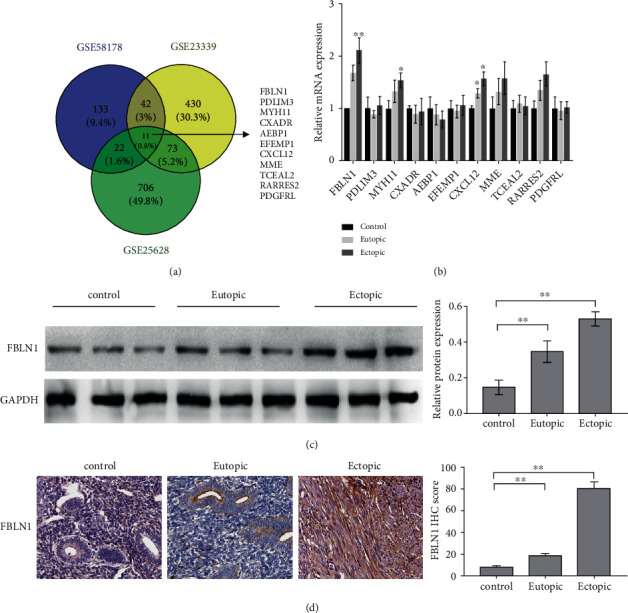
FBLN1 expression was upregulated in eutopic and ectopic endometrial tissues with EMS. (a) The datasets of GSE58178, GSE23339, and GSE25628 were downloaded from the GEO database, and the differentially expressed genes were identified using Venn diagram analysis. (b) qPCR analysis of 11 common dysregulated genes (FBLN1, PDLIM3, MYH11, AEBP1, EFEMP1, CXCL12, TCEAL2, RARRES2, PDGFRL, CXADR, and MME) in ectopic endometrial tissues (*n* = 3), paired eutopic endometrial tissues (*n* = 3), and normal endometrial tissues (*n* = 3). (c) Western blot analysis of FBLN1 protein expression in ectopic endometrial tissues (*n* = 3), paired eutopic endometrial tissues (*n* = 3), and normal endometrial tissues (*n* = 3). (d) IHC analysis of FBLN1 level in ectopic endometrial tissues (*n* = 3), paired eutopic endometrial tissues (*n* = 3), and normal endometrial tissues (*n* = 3). The representative IHC image was shown in the left panel, and FBLN1 IHC score was shown in the right panel. ^∗^*p* < 0.05, ^∗∗^*p* < 0.01, and ^∗∗∗^*p* < 0.001.

To investigate the molecular mechanisms involved in EMS, the datasets of GSE58178, GSE23339, and GSE25628 were downloaded from GEO (https://www.ncbi.nlm.nih.gov/gds/?), and differentially expressed genes in these datasets were separately identified using GEO2R tool (log | FC | >1.5 and *p* < 0.05). The results from Venn diagram analysis (Venny 2.1 tool, https://bioinfogp.cnb.csic.es/tools/venny_old/index.html) showed that 9 genes (FBLN1, PDLIM3, MYH11, AEBP1, EFEMP1, CXCL12, TCEAL2, RARRES2, and PDGFRL) were concurrently upregulated and 2 genes (CXADR and MME) were concurrently downregulated in ectopic endometrial tissues compared with normal endometrial tissues (Figure 1(a)). To validate this, eutopic and ectopic endometrial tissues of patients with EMS were collected and the expression of these genes was assessed using qPCR. As shown in Figure 1(b), the mRNA expression of FBLN1, MYH11, and CXCL12 was upregulated significantly in eutopic and ectopic endometrial tissues of patients with EMS compared with normal endometrial tissues. Among the three genes, we investigated FBLN1 expression pattern and biological function in EMS because FBLN1 was the most significantly upregulated gene.  Figure 1(c) shows that FBLN1 protein expression was increased in eutopic and ectopic endometrial tissues of patients with EMS compared with controls. IHC analysis also revealed that FBLN1 protein level was upregulated in eutopic and ectopic endometrial tissues (Figure 1(d)). These results indicate that the aberrant expression of FBLN1 might be correlated with EMS.

### 3.2. FBLN1 Increased ESC Viability and Facilitated ESC Migration

**Figure 2:**
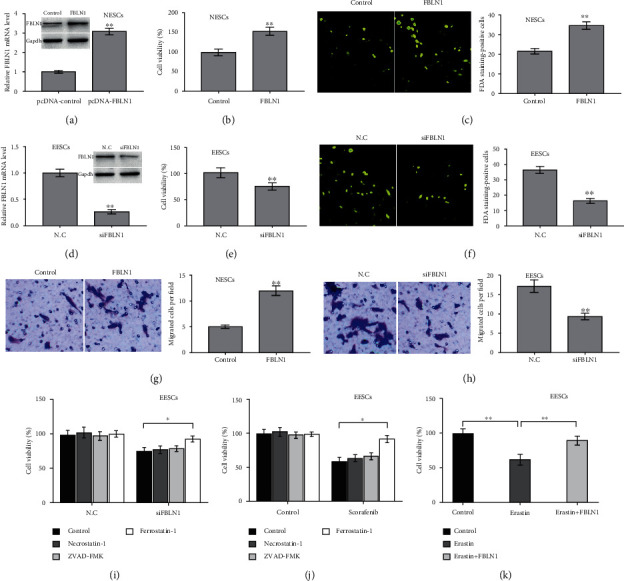
FBLN1 increased ESC viability and facilitated ESC migration. (a) Normal ESCs (NESCs) were treated with pcDNA-FBLN1 (or pcDNA control) for 48 h, and then, qPCR and western blot analysis were carried out to assess the FBLN1 protein expression in NESCs. After FBLN1 overexpression in NESCs, (b) CCK-8 assay and (c) FDA staining were carried out to assess cell viability. (d) Ectopic ESCs (EESCs) were treated with siFBLN1 (or negative control (N.C)) for 48 h, and then, qPCR and western blot analysis were carried out to assess the FBLN1 protein expression in EESCs. After FBLN1 silencing in EESCs, (e) CCK-8 assay and (f) FDA staining were carried out to assess cell viability. (g) After FBLN1 overexpression in NESCs, transwell migration assay was carried out to assess NESC migration. Scan bar = 50 *μ*m. Magnification ×20. (h) After FBLN1 silencing in EESCs, transwell migration assay was carried out to assess NESC migration. Scan bar = 50 *μ*m. Magnification ×20. (i) EESCs were treated with ZVAD-FMK (10 *μ*M), necrostatin-1 (10 *μ*M), and ferrostatin-1 (1 *μ*M) in the presence or absence of siFBLN1, and then, CCK-8 assay was carried out to assess EESC viability. (j) EESCs were treated with ZVAD-FMK (10 *μ*M), necrostatin-1 (10 *μ*M), and ferrostatin-1 (1 *μ*M) in the presence or absence of sorafenib, and then, CCK-8 assay was carried out to assess EESC viability. (k) EESCs were treated with erastin (10 *μ*M) in the presence or absence of FBLN1 overexpression, and then, CCK-8 assay was carried out to assess EESC viability. *p* < 0.05 and ^∗∗^*p* < 0.01.

Given that FBLN1 expression was higher in ectopic endometrial tissues than in normal endometrial tissues, FBLN1 was overexpressed in normal ESCs (NESCs) and knocked down in ectopic ESCs (EESCs), and then, the effect of FBLN1 on NESC and EESC viability was assessed. Figures 2(a) and 2(b) show that FBLN1 overexpression increased NESC viability. The results from FDA staining also exhibited the effect of FBLN1 on enhancing cell viability (Figure 2(c)). On the contrary, FBLN1 silencing in EESCs repressed cell viability (Figures 2(d)–2(f)). Moreover, FBLN1 overexpression facilitated NESC migration, whereas FBLN1 silencing repressed EESC migration (Figures 2(g) and 2(h)). Interestingly, the CCK-8 assay revealed that FBLN1 silencing-mediated cell death of EESCs was blocked significantly by ferrostatin-1 (Fer-1, a specific inhibitor of ferroptosis) but not ZVAD-FMK (a specific inhibitor of apoptosis) and necrostatin-1 (a specific inhibitor of necroptosis) (Figure 2(i)). Sorafenib was reported as an effective drug to control EMS progression [28], and sorafenib could facilitate cell death by inducing ferroptosis [29]. Consistent with previous studies, Figure 2(j) shows that sorafenib-induced EESC death was blocked markedly by Fer-1, but not ZVAD-FMK and necrostatin-1. Erastin (a specific activator of ferroptosis) treatment facilitated EESC death, but FBLN1 overexpression repressed significantly the effect of erastin on EESC death (Figure 2(k)). These data demonstrate that FBLN1 increased ESC viability and migration, at least in part by regulating ferroptosis.

### 3.3. FBLN1 Inhibited EESC Ferroptosis

**Figure 3:**
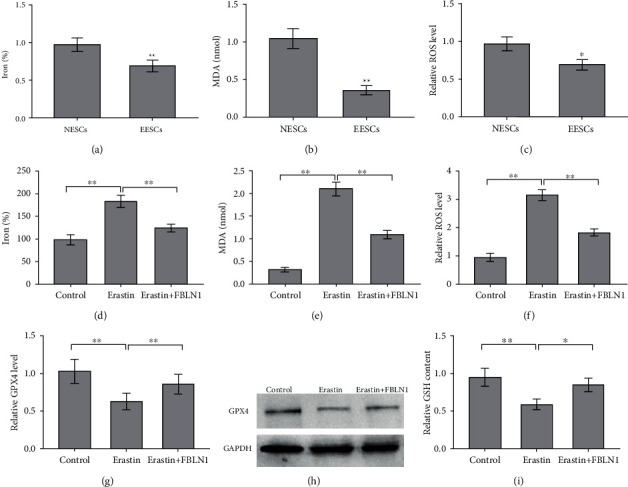
FBLN1 inhibited the ferroptosis of ESCs. The levels of (a) Fe^2+^ and (b) MDA and (c) ROS were assessed using the indicated kits in NESCs and EESCs. The levels of (d) Fe^2+^ and (e) MDA and (f) ROS were assessed using the indicated kits in EESCs after erastin (10 *μ*M) treatment in the presence or absence of FBLN1 overexpression. The levels of (g and h) Gpx4 and (i) GSH were assessed using the indicated kits in EESCs after erastin (10 *μ*M) treatment in the presence or absence of FBLN1 overexpression. ^∗^*p* < 0.05 and ^∗∗^*p* < 0.01.

To investigate the role of FBLN1 in ESC ferroptosis, ferroptosis signaling was first evaluated in NESCs and EESCs. Figures 3(a)–3(c) show that the levels of Fe^2+^, MDA, and ROS levels were decreased significantly in EESCs compared with NESCs, indicating a decreased ferroptosis signaling in EESCs. Furthermore, FBLN1 overexpression repressed erastin-induced increase of Fe^2+^, MDA, and ROS levels in EESCs (Figures 3(d)–3(f)). FBLN1 overexpression also blocked the effect of erastin on inhibiting the Gpx4 expression and GSH content (Figures 3(g)–3(i)). These results verify that upregulated FBLN1 repressed the signaling of EESC ferroptosis.

### 3.4. FBLN1 Inhibited ESC Ferroptosis, Viability, and Migration via Increasing the Stability of EFEMP1 Protein

**Figure 4:**
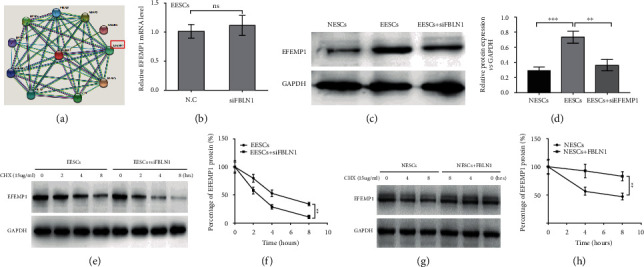
FBLN1 increased the stability of EFEMP1 protein. (a) STRING tool (https://string-db.org/) was applied to predict the proteins that may interact with FBLN1. (b) EESCs were treated with siFBLN1, and then, qPCR analysis was carried out to assess the mRNA level of EFEMP1 in EESCs. (c, d) EESCs were treated with siFBLN1 (or N.C), and then, western blot analysis was carried out to assess the protein level of EFEMP1 in EESCs and NESCs. (e) EESCs were treated with siFBLN1 (or N.C) in the presence of CHX, and then, western blot analysis was carried out to assess the EFEMP1 protein level at 0, 2, 4, and 8 h. (f) Relative quantitative analysis of western blot showed in (e). (g, h) NESCs were treated with pcDNA-FBLN1 (or control) in the presence of CHX, and then, western blot analysis was carried out to assess the EFEMP1 protein level at 0, 2, 4, and 8 h.

**Figure 5:**
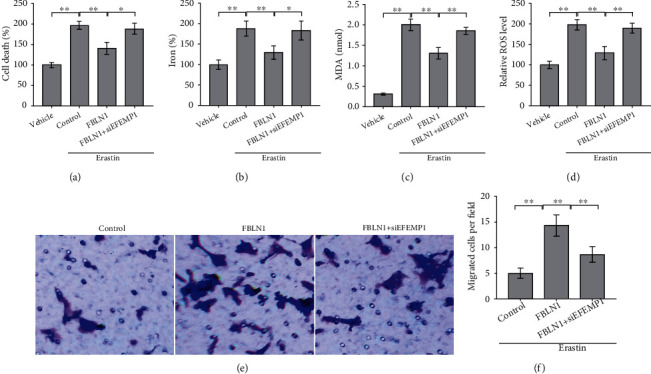
FBLN1 inhibited EESC ferroptosis, viability, and migration via increasing the stability of EFEMP1 protein. The levels of (a) Fe^2+^ and (b) MDA and (c) ROS were assessed using the indicated kits in erastin (10 *μ*M)-treated EESCs after FBLN1 overexpression in the presence or absence of EFEMP1 silencing. (d) The cell death was assessed using CCK-8 assay in erastin (10 *μ*M)-treated EESCs after FBLN1 overexpression in the presence or absence of EFEMP1 silencing. (e, f) Erastin (10 *μ*M)-treated EESCs were transfected with pcDNA-FBLN1 in the presence or absence of EFEMP1 silencing, and then, transwell migration analysis was carried out to assess EESC migration silencing. ^∗^*p* < 0.05 and ^∗∗^*p* < 0.01.

To explore the molecular mechanism involved in FBLN1-regulated ESC ferroptosis, we carried out a protein-protein interaction network analysis by STRING (https://string-db.org/cgi/input). As shown in Figure 4(a), one of the 11 concurrently differentially expressed genes in GSE58178, GSE23339, and GSE25628 datasets (shown in Figure 1(a)), EFEMP1, was a potential protein interacting with FBLN1. Therefore, EFEMP1 was selected for further validation. Although the mRNA level of EFEMP1 was not differential between normal endometrium and eutopic endometrium (Figure 1(b)), the protein level of EFEMP1 was upregulated in EESCs compared with NESCs (Figures 4(c) and 4(d)). Similarly, the mRNA level of EFEMP1 was unchanged in EESCs after FBLN1 silencing (Figure 4(b)), but FBLN1 silencing resulted in a significant decrease of EFEMP1 protein level (Figures 4(c) and 4(d)), indicating that FBLN1 regulates the stability of EFEMP1 protein. The cycloheximide chase assay showed that FBLN1 silencing in EESCs decreased significantly the half-life of EFEMP1 protein (Figures 4(e) and 4(f)), whereas FBLN1 overexpression increased significantly the half-life of EFEMP1 protein in NESCs (Figures 4(g) and 4(h)). Functionally, EFEMP1 silencing blocked significantly the effect of FBLN1 on repressing EESC ferroptosis, as evidenced by assessing the level of Fe^2+^ (Figure 5(a)), MDA (Figure 5(b)), and ROS (Figure 5(c)). As a result, EFEMP1 silencing partially destroyed the effect of FBLN1 on facilitating EESC viability (Figure 5(d)) and migration (Figures 5(e) and 5(f)). Taken together, the current results demonstrate that upregulated FBLN1 increased ESC viability and migration by regulating EFEMP1-dependent ferroptosis in EMS.

## 4. Discussion

At present, the incidence of EMS is increasing year by year, but its pathogenesis is still unclear. In the present study, we demonstrated that (i) FBLN1 expression was upregulated in eutopic and ectopic endometrial tissues with EMS; (ii) FBLN1 increased ESC viability and migration; (iii) FBLN1 decreased the ferroptosis of ESCs; and (iv) FBLN1 repressed ESC ferroptosis, viability, and migration via increasing the stability of EFEMP1 protein.

FBLN1 is a member of family of extracellular glycoproteins that regulates cell morphology, cell migration, and cell interaction with extracellular matrix [30, 31]. The tumor-suppressing role of FBLN1 has been described in many types of cancers like gastric carcinoma, prostate cancer, breast cancers, and ovarian cancers [32]. EMS is an estrogen-dependent gynecological condition influenced by multifarious environmental and genetic factors [33]. The increase of estrogen activity is the main hormonal substrate of EMS and is correlated with inflammation [34]. Previous studies have demonstrated that the expression of FBLN1 is regulated by estrogen [35]. Moreover, the association of aberrant expression of FBLN1 with EMS has been verified [35]. However, the biological role of FBLN1 in EMS progression remains unclear. In the current study, the results from CCK-8, FDA staining, and transwell analysis showed that FBLN1 overexpression increased NESC viability and migration, whereas FBLN1 silencing in EESCs repressed cell viability and migration. These data demonstrate for the first time that upregulation of FBLN1 facilitates EMS progression. Furthermore, the present data showed that sorafenib-induced cell death of EESCs was blocked markedly by Fer-1, but not ZVAD-FMK and necrostatin-1, indicating that FBLN1 increased ESC viability and migration, at least in part by regulating ferroptosis. Given that FBLN1 silencing in EESCs repressed cell viability and the levels of Fe^2+^, MDA, and ROS were significantly decreased in EESCs compared with NESCs, we only assessed the effect of ferrostatin-1, ZVAD-FMK, and necrostatin-1 on EESCs after FBLN1 silencing.

Ferroptosis is an iron-dependent form of nonapoptotic cell death [36]. The accumulation of ROS in cells is one of the direct causes of ferroptosis [10]. Ng et al. showed (i) that a major defect in EMS is abnormal eutopic endometrium characterized by resistance to ferroptosis, which allows cells spread through retrograde menstrual spreading to survive, implant, and establish endometrial lesions in the peritoneal cavity, and (ii) that the dysregulation of iron homeostasis may be vital to the subsequent pathophysiology of endometrial pathologies accompanied by local iron overload and inflammation [8]. In our study, the role of FBLN1 in ESC ferroptosis was investigated. The levels of Fe^2+^, MDA, and ROS were decreased significantly in EESCs compared with NESCs; FBLN1 overexpression repressed erastin-induced increase of Fe^2+^, MDA, and ROS levels and blocked the effect of erastin on Gpx4 and GSH levels. The present results were partly inconsistent with those previously reported, in which ROS levels are high in endometriosis tissue and follicular fluid [37–39]. The reason why oxidative stress activity was decreased in EESCs will be investigated in the future.

To explore the molecular mechanism underlying the regulation of FBLN1 for ESC ferroptosis, the target proteins that may act with FBLN1 were predicted through STRING website. EFEMP1 was found to be associated with EMS and FBLN1. Although the mRNA level of EFEMP1 was not differential between normal endometrium and eutopic endometrium, FBLN1 silencing resulted in a significant decrease of EFEMP1 protein level, indicating that FBLN1 regulates the stability of EFEMP1 protein. Functionally, EFEMP1 silencing blocked significantly the effect of FBLN1 on repressing EESC ferroptosis, as evidenced by assessing the level of Fe^2+^, MDA, and ROS. EFEMP1 inhibition destroyed the effect of FBLN1 on repressing ESC death and facilitating ESC migration. Taken together, the current results demonstrate that upregulated FBLN1 increased ESC viability and migration by regulating EFEMP1-dependent ferroptosis in EMS.

## Data Availability

The data used to support the findings of this study are available from the corresponding author upon request.

## Abbreviations

EMS: Endometriosis
GEO: Gene Expression Omnibus
ESCs: Endometrial stromal cells
MDA: Malondialdehyde
GPX4: Glutathione peroxidase 4
GSH: Glutathione
EFEMP1: EGF-containing fibulin-like extracellular matrix protein 1.
